# Peer-support to increase uptake of screening for diabetic retinopathy: process evaluation of the DURE cluster randomized trial

**DOI:** 10.1186/s41182-019-0188-z

**Published:** 2020-01-06

**Authors:** Nyawira Mwangi, Covadonga Bascaran, Jacqueline Ramke, Mathew Kipturgo, Min Kim, Mark Ng’ang’a, Stephen Gichuhi, Dorothy Mutie, Consuela Moorman, Lawrence Muthami, Allen Foster

**Affiliations:** 1 London School of Hygiene and Tropical Medicine, London, England; 2 Kenya Medical Training College, Nairobi, Kenya; 3 University of Auckland, Auckland, New Zealand; 4 Kerugoya County Referral Hospital, Kerugoya, Kenya; 5 University of Nairobi, Nairobi, Kenya; 6 Oxford University NHS Trust, Oxford, UK; 7 Kenya Medical Research Institute, Nairobi, Kenya

**Keywords:** Diabetes, Diabetic retinopathy, Peer support, Cluster-randomized clinical trial, Consolidated Framework of Implementation Research (CFIR), Process evaluation

## Abstract

**Background:**

There is limited evidence on how implementation of peer support interventions influences effectiveness, particularly for individuals with diabetes. We conducted a cluster randomized controlled trial to compare the effectiveness of a peer-led health education package versus usual care to increase uptake of screening for diabetic retinopathy (DR).

**Methods:**

Our process evaluation used a mixed-method design to investigate the recruitment and retention, reach, dose, fidelity, acceptability, and context of implementation, and was guided by the Consolidated Framework for Implementation Research (CFIR). We reviewed trial documents, conducted semi-structured interviews with key informants (*n* = 10) and conducted four focus group discussions with participants in both arms of the trial. Three analysts undertook CFIR theory-driven content analysis of the qualitative data. Quantitative data was analyzed to provide descriptive statistics relevant to the objectives of the process evaluation.

**Results:**

The trial had positive implementation outcomes, 100% retention of clusters and 96% retention for participants, 83% adherence to delivery of content of group talks (fidelity), and 78% attendance (reach) to at least 50% (3/6) of the group talks (dose). The data revealed that intervention characteristics, outer setting, inner setting, individual characteristics, and process (all the constructs of CFIR) influenced the implementation. There were more facilitators than barriers to the implementation. Facilitators included the relative advantage of the intervention compared with current practice (intervention characteristics); awareness of the growing prioritization of diabetes in the national health policy framework (outer setting); tension for change due to the realization of the vulnerability to vision loss from DR (inner setting); a strong collective sense of accountability of peer supporters to implement the intervention (individual characteristics); and regular feedback on the progress with implementation (process). Potential barriers included the need to queue at the eye clinic (intervention characteristic), travel inconveniences (inner setting), and socio-political disruption (outer setting).

**Conclusions:**

The intervention was implemented with high retention, reach, fidelity, and dose. The CFIR provided a valuable framework for evaluating contextual factors that influenced implementation and helped to understand what adaptations may be needed during scale up.

**Trial registration:**

Pan African Clinical Trials Registry: PACTR201707002430195 registered 15 July 2017

## Background

Early detection of diabetic retinopathy (DR) poses a significant medical and public health challenge, particularly because DR is asymptomatic until the advanced stages. The benefits of regular screening have been documented [1], but uptake remains low for people living with diabetes (PLWD) in settings without systematic DR screening programs [2]. Diabetes Support Groups (DSGs) provide an opportunity for demand-side interventions to increase attendance at screening and confront this inequity [3]. However, there is need for evidence on the factors that influence implementation and outcomes of DR interventions involving DSGs.

The DURE (Uptake of Retinal Examination in Diabetes mellitus) trial was a 6-month pragmatic cluster randomized controlled trial (cRCT) to evaluate the effectiveness of a complex intervention to promote screening for diabetic retinopathy among members of DSGs in Kirinyaga County, Kenya. The DURE trial interventions have been described in detail [3]. Briefly, the trial compared the proportion of PLWD who attended screening in seven DSGs that received the intervention with seven “usual care” DSGs that did not receive the intervention. The intervention consisted of (i) training of peer supporters; (ii) monthly group talks at the DSGs by peer supporters and referral of PLWD to the eye clinic; (iii) monthly individual reminders to PLWD (by peer supporters) to attend screening the eye clinic; and (iv) weekly telephone support to peer supporters from the research team.

The intervention was developed in accordance with the guidelines of the Medical Research Council (MRC) framework for complex interventions [4]. These guidelines recommend using appropriate theory to develop interventions that address the barriers to behavior change. Our formative research identified several barriers to uptake of DR screening [2]. Self efficacy is a strong precursor to behavior change [5, 6] including attendance to screening [7]. Based on the self-efficacy theory, we hypothesized that an intervention that increases self-efficacy can decrease the perceived barriers to attendance to screening. The theory proposes four methods of changing self-efficacy in order to change behavior: providing mastery experiences (e.g., recalling previous screening for other diabetes complications); vicarious learning (e.g., from hearing experiences of peers who have had DR screening); verbal persuasion (of the need for screening); and addressing psychological and affective states (such as anxiety about taking a screening exam) [6]. The theory-driven conceptual framework of intervention effect is illustrated in the protocol [3].

The MRC framework [4] emphasizes four key phases of interventions: intervention development; feasibility and piloting; implementation; and the evaluation of both outcomes and process. In this paper, we describe the results of the process evaluation. Process evaluation is a study which aims to understand the functioning of an intervention, by examining implementation, mechanisms of impact, and contextual factors [8]. The conduct of process evaluations alongside RCTs has been recommended, because they give insight into the “black box” of health care interventions; facilitate the interpretation of the findings; explain why, for whom and how a complex intervention had a particular impact; and determine whether a complex intervention should be scaled up or modified for other contexts [9, 10]. Process evaluations are particularly important in cRCTs, because of the potential for between-cluster differences that need to be understood [10, 11].

**Fig. 1:**
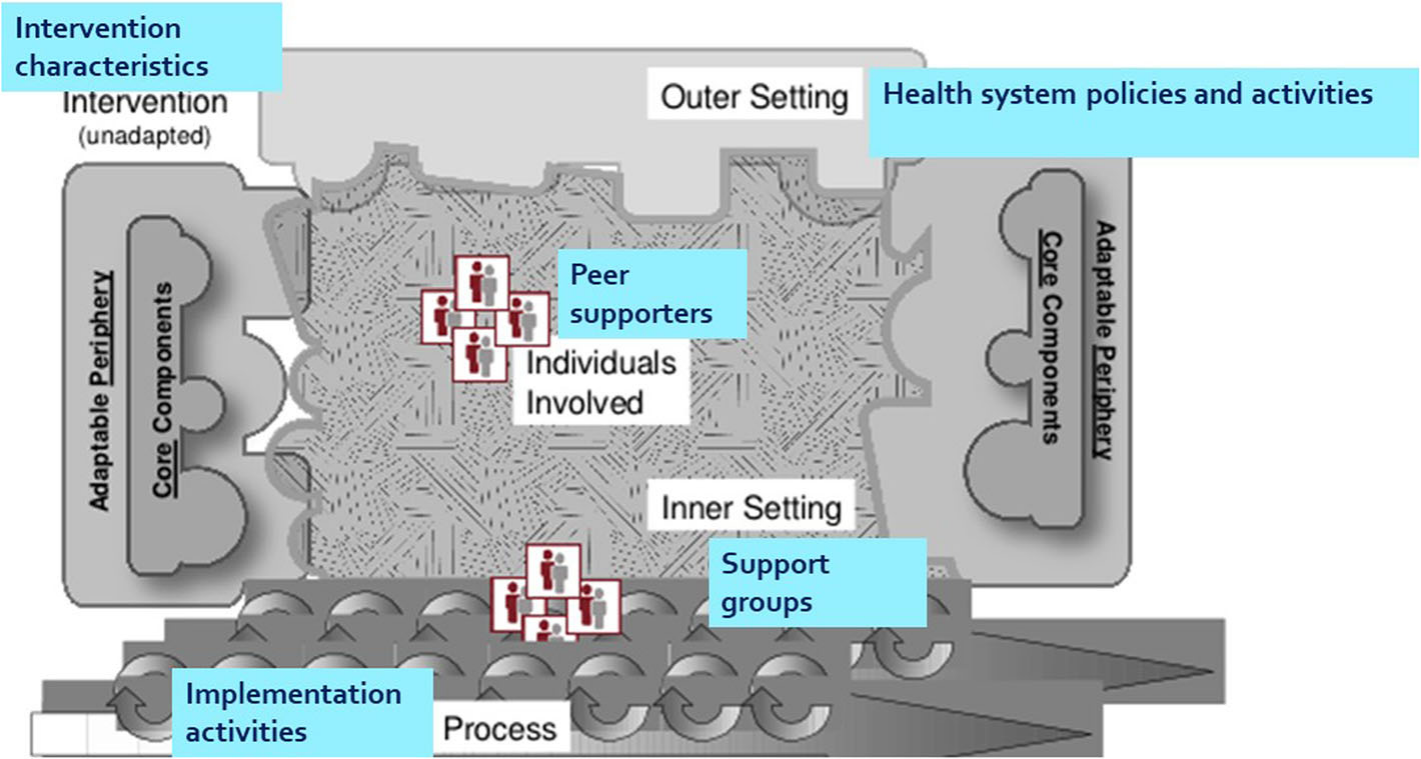
Consolidated Framework for Implementation Research as it relates to this trial (adapted from Dramschroder et al, 2009 [13])

Theory-driven process evaluation necessitates that the designers make the theory explicit and then use it to identify how the intervention leads to the outcomes [12]. The Consolidated Framework for Implementation Research (CFIR) is a is a meta-theoretical framework that synthesizes constructs from multiple theories on implementation of interventions, in order to explain what works and why across multiple contexts [13]. The CFIR outlines five major factors that influence implementation of interventions: the characteristics of the intervention, the inner setting, the outer setting, the individuals involved, and the process of implementation (Table 1). By applying this framework to the process evaluation for this cRCT (Fig. 1), we aimed to (1) understand the determinants for the outcomes of the DURE intervention in Kirinyaga and (2) examine the context of implementation in terms of the intervention’s recruitment and retention, reach, fidelity, dose, and acceptability (Table 2).

**Table 1:** Constructs of the Consolidated Framework for Implementation Research

Construct	How the construct relates to the DURE trial
Characteristics of the intervention**—**core components and adaptable components	Intervention characteristics (adaptability complexity, relative advantage) can influence whether the intervention is adopted
Inner setting—structural, political, and cultural context that directly affects the implementation	The context of the DSGs and the eye health system in Kirinyaga can influence how participants experience the intervention
Outer setting—broader economic, political and social context	Broader political, economic, health policies, priorities, resources, incentives, and governance may impact trial activities
Characteristics of individuals—people responsible for delivering the intervention (peer supporters)	Training, knowledge, perceptions, motivation, and leadership of peer supporters can influence extent of implementation of the intervention
Implementation process—the activities involved in planning, engaging, execution, and evaluation of implementation process	The involvement of stakeholders in the planning, execution, and evaluation of progress of the trial may influence the acceptability of delivery and reception of the intervention

*DSG* Diabetes Support Group, *DURE* Uptake of Retinal Examination in Diabetes study

**Table 2:** Measures for the level of implementation

Implementation measure	Questions related to DURE study	Quantitative indicator(s)—compared with target
Recruitment and retention	How successful were the recruitment and retention procedures?	-Proportion of DSGs that agreed to participate (%) -Proportion of participants invited who agreed to participate (%) -Peer educators recruited, trained and retained (*n*) -Retention rate of clusters and participants in the study (%)
Reach	What proportion of the intended audience was exposed to the intervention?	-People who were referred (*n*) -People who received individual reminders (*n*) -People who attended group meetings (*n*)
Dosage delivered	What percentage of interventions was delivered most/least successfully by implementers?	-Group talks delivered (*n*) -Referrals made (*n*) -Individual reminders given(*n*)
Dose received	What percentage of the intervention was received most/least successfully by the target audience?	-Proportion of group sessions attended by each participant (%) -Referrals given (*n*)
Fidelity	How much of the intervention was delivered as intended (adherence)? What parts were not delivered?	-Adherence to content of group talks -Adherence to frequency of group talks
Acceptability	How acceptable is the intervention for current and future implementation?	Acceptance of the intervention by peer supporters and participants Willingness of stakeholders to scale up the intervention in future

*DSG* Diabetes Support Group, *DURE* Uptake of Retinal Examination in Diabetes study

## Methods

### Ethics

The DURE trial and its process evaluation has ethics approval from the research ethics committees of the London School of Hygiene and Tropical Medicine in London and the African Medical Research Foundation in Nairobi. Trial registration: Pan African Clinical Trials Registry: PACTR201707002430195.

### Setting

The setting of the study is described elsewhere [3]. Briefly, Kirinyaga county is a rural agrarian county in Central Kenya. The prevalence of diabetes in Kenya is estimated to be 2% in the population 18–64 years [14]. An estimated 40% of the PLWD in Kirinyaga are members of DSGs, and a health system assessment by our research group found that only 7% of them have had an annual DR screening exam as recommended. DSGs have monthly meetings in the community led by peer supporters, where they measure the weight, blood sugar, and blood pressure of attenders. They also engage in other activities relevant to health promotion and advocacy. At the time of the DURE study, there were 16 DSGs active in Kirinyaga. Two of them participated in the pilot trial, while the other 14 were recruited into the main trial.

Two peer supporters were recruited in each DSG (1 male, 1 female), as per the eligibility criteria in Table 3, and none of them had previously delivered an eye health intervention. They received two days of training using a curriculum developed through the process described in Additional file 1. The content of the training and the key messages that the peer supporters delivered to participants are described in the protocol [3]. An allowance was provided to peer supporters for telephone communication with participants, but no other financial incentives were given. Weekly telephone calls between the principal investigator and peer supporters were carried out to share progress, build a sense of belonging, and address any challenges emerging during the program.

**Table 3:** Eligibility criteria for participants and peer-supporters

Criterion	Participants	Peer-supporters
Age > 18 years	√	√
Member of a diabetes support group	√	√
Will reside in the county for the next 12 months	√	√
Has a mobile phone	√	√
Willing to participate in the study	√	√
Had not had a screening exam in the last 12 months	√	×
Has had a screening exam in the preceding 12 months	×	√
Willing to be a peer-supporter	N/A	√
Willing to commit 2 days for training	N/A	√
Willing to commit many hours to peer-support work	N/A	√
Fluent in Kikuyu or Kiswahili	N/A	√
Already attending DR screening	×	√
Already receiving treatment for DR	×	×
Has a debilitating illness	×	×

“*√*” indicates participants or peer-supporters included in the study

“×”indicates participants or peer-supporters excluded in the study

*N/A* Not applicable

The county has a well-equipped eye clinic at the Kerugoya county referral hospital. Patients at the clinic are attended on a walk-in basis. There were four eye health workers (one ophthalmologist, three ophthalmic clinical officers) in the county during the study period. Guidelines for screening and management of diabetic retinopathy were launched at the national level 3 months before the start of the main trial, and were also being implemented in Kirinyaga county [15].

### Design

This is a mixed-methods process evaluation of a cRCT. We used the CFIR to guide the process because it is comprehensive and can be used to develop the evaluation tools, guide the content analysis, and aid interpretation of findings [16].

### Data collection

Data on recruitment and retention, fidelity, reach, and dose were collected routinely during the trial activities and collated through document review. Peer supporter training records provided information on attendance and content of training. Trial registers provided data on recruitment, while DSG meeting attendance registers captured participants attendance and retention, as they provided the date of the meeting and the list of attendees. DSG meeting minutes, peer supporter diaries, and the research team’s activity logs, and field notes contained detailed information on the intervention activities, personnel involved, duration, frequency, and resources used.

We studied the sample interview questions available on http://cfirguide.org/ and tailored our data collection tools to gather information relevant to the DURE study. These questions related to the stakeholders’ perception of the intervention and how it worked/did not work.

Ten interviews were conducted with purposively selected key informants to represent recipients, implementers, administrators, and policy-makers. Key informants were recruited until data saturation was reached. Face to face interviews were conducted by the first author in English at locations convenient to the key informant, using a semi-structured interview schedule. Interviews lasted 30–60 min and were captured through field notes.

We conducted four focus group discussions in community settings with 7 participants in the intervention arm who did not take up screening; 7 participants in the intervention arm who took up screening; 8 participants from the control arm; and 7 peer supporters. Focus group discussions were conducted in the Kikuyu language by the first author and two research assistants who were considered culturally appropriate but not involved in the trial implementation. Discussions were audio-recorded, transcribed, and translated into English.

### Data analysis

Quantitative data on recruitment and retention, reach, fidelity, and dose were analysed for descriptive summary statistics.

A thematic content analysis of qualitative data was undertaken based on the different constructs and sub-constructs of the CFIR: (1) Intervention characteristics (e.g., acceptability of the intervention, compatibility with existing DSG programs, relative advantages, or disadvantages of the intervention, and suggested adaptations); (2) Outer setting (e.g., perceived role of the Ministry of Health (MoH) policies and guidelines in driving which services were implemented); (3) Inner setting (e.g., perceptions about organizational factors within DSGs and the eye clinic, that might have affected the implementation); (4) Individual characteristics of peer supporters who delivered the intervention (e.g., knowledge, attitudes, self-efficacy about their role); and (5) Implementation processes (e.g., planning, engagement, and execution factors that may have affected the delivery and reception of the DURE intervention). Using the CFIR as a template framework, the principal investigator and two other analysts (1 male, 1 female) read the transcripts, coded the data independently, and grouped the codes into themes. Analysis began as soon as the first interview was completed, and then proceeded concurrently with data collection until data saturation was reached. The analysts reviewed the codes iteratively to check for potential biases, and to verify the emerging themes. Discrepancies between coders were resolved through discussion and review of the original transcripts.

Data (qualitative and quantitative) from all sources were organized under the respective constructs and sub-constructs to facilitate triangulation and to identify which factors affected the acceptability, recruitment, retention, reach, fidelity, and dose of implementation.

The first author had undertaken training on implementation research and clinical trials, and had expertise on the technical content of the intervention. All the analysts had skills in quantitative and qualitative research methods.

## Results

### How was the intervention implemented?

#### Recruitment and retention

**Fig. 2:**
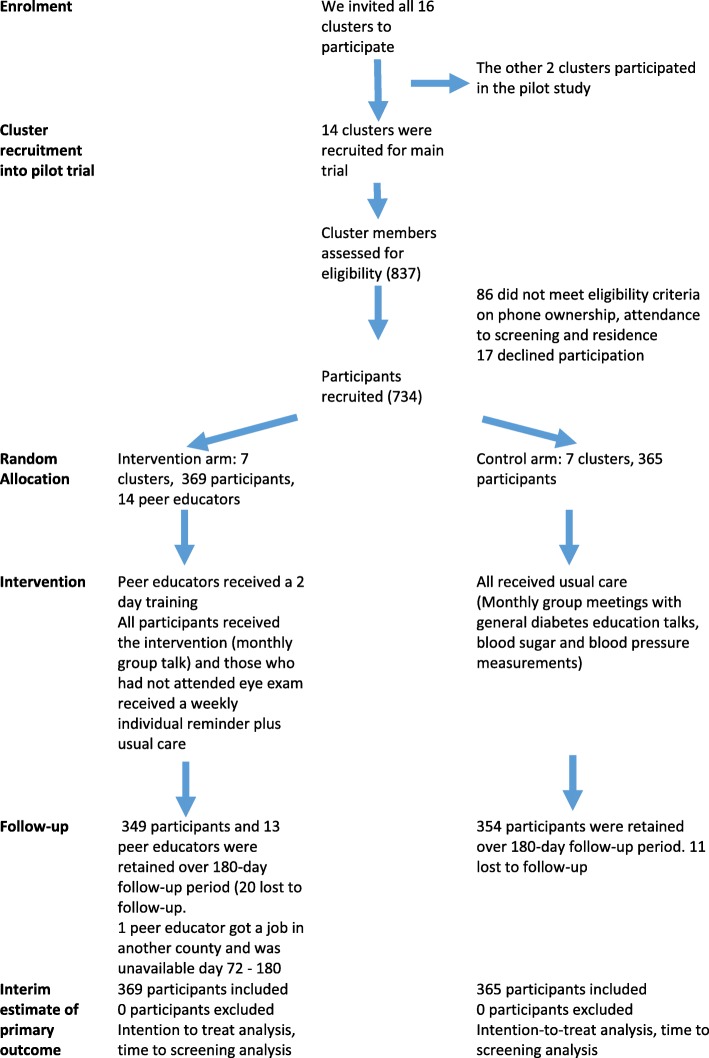
Flow diagram for the trial

All the 16 DSGs in the county accepted to participate in the trial (2 in the pilot trial and 14 in the main trial). All clusters were retained throughout the study. Of 837 members of DSGs approached to participate the main trial, 86 did not meet eligibility criteria (Fig. 2), and 17 did not consent, thus 734 participants were recruited and participated in the trial. Of these, 31 (4.2%) were lost to follow-up during the trial (95.8% follow-up rate rate). The 14 peer supporters (age range was 29–58 years) received the training program. All had at least secondary education, with three having achieved a tertiary level qualification, although this was not a requirement. One peer supporter was absent from day 72 as another DSG project that was being implemented a different county contracted him for their team. The remaining 13 actively participated in the DURE trial until the end of the trial. The research team maintained weekly contact with peer supporters and attended some of the DSG meetings, which minimized the likelihood of loss to follow-up or missing data. Recruitment procedures had already been tested in the pilot trial [17].

#### Reach

Out of 369 PLWD recruited into the intervention arm, 92% attended the first group talk, while 72% attended all six talks. Seventy-eight percent attended three or more talks (50%), and this ranged from 68% in the most rural DSG to 88% in urban DSGs. One hundred percent of the participants were referred for the screening examination and were issued with a referral card. Peer supporters also gave monthly telephone reminders to those who had not yet taken the screening exam. Seventy-four percent of participants received at least one telephone reminder to attend screening.

#### Dose delivered and dose received

The 14 peer supporters attended the training. During the 6 months of the study, peer supporters in the seven DSGs in the intervention arm delivered a group talk each month (total 42 talks, 100% of planned talks were delivered). We found that 34/42 (81%) meetings had an attendance of ≥ 80% of the trial participants.

Of the targeted 369 telephone reminders in the first month, 273 (74%) telephone reminders were made as some of the participants took the screening exam immediately after the first group talk and before the telephone reminders. Forty-eight percent of participants received a reminder each month (six reminders in total) over the 6 months trial period. There were frequent reports of participants being unreachable on phone, as the phones were switched off, or they had changed the number. In such cases, the peer supporter arranged a personal visit to the participant to give a face-to-face reminder (this was a local adaptation of the intervention). During the first two months of implementation, peer supporters from two different DSGs made weekly calls to the principal investigator to seek feedback (in addition to the weekly calls that all peer supporters received from the principal investigator). This was a further form of intervention adaptation; these two DSGs had the highest rates of implementation fidelity.

#### Fidelity

There was 100% adherence to the frequency of the group talks (one group talk every month). However there were occasional changes on the actual date of the group talk each month (due to other communal activities), such that the intervals between the group talks were not constant. Adherence to content of group talks met the required threshold in 83% of the group talks. Some 43 participants could not be reached on telephone at least once during the trial, and the peer supporters gave them a reminder through a face-to-face communication as mentioned above. We monitored adaptations, which we interpreted as evidence of ownership and adaptation to meet contextual needs.

#### Acceptability

The acceptance of the intervention in the study population was high, given the high retention in the study. The intervention was perceived as beneficial and acceptable to participants because it was bridging information gaps.

*I am happy you people are coming here to us, because we are benefiting. Although I have not yet gone [for screening], I now know that I should not wait to have problems with my vision and I know where I should go [for screening]* (*FGD participant*)

There was satisfaction with the study procedures among the participants. This was not surprising as we had tested these with a pilot study [17]. We had anticipated participants to be uncomfortable with temporary blurring of vision due to dilating drops, but it was not perceived to be a significant problem because they were forewarned about it.

*The difficult seeing after the medicine in the eye…it was not as serious as I expected,… it was not like you could not see at all…, and by the time I was going home I could see very well* (*FDG participant*)

All 10 key informants were willing to scale up the intervention to other counties in future, using the DURE tools such as the PS training curriculum and the community entry mechanism.

*The DURE curriculum for the peer supporter training is very comprehensive and easy to follow, and we want to formally adopt it in our peer supporter manual, so that we can use it in our routine training of peer supporters* (*KDDA national representative*)

Eye care workers accepted to conduct the screening for DR, even though at first they were concerned about a sudden increase in the screening workload.

*Initially we had concern that this might increase the workload, but we found that this was only a temporary effect as many participants came at the same time for the screening, which is unlikely to happen in the repeat screening visits. It is good to see the participants asking for the screening… they already have the information about it* (*eye health worker*)

### How was the intervention experienced?

Table 4 shows the five CFIR constructs, the sub-constructs identified within them and examples of the related quotes.

**Table 4:** Quotes on CFIR constructs

CFIR construct/sub-construct	Sample quotes
Intervention characteristics	
Relative advantage	This is more effective than leaving it to health workers from the hospital to go to the community to educate the PLWD, for that is not always feasible (MoH national representative) The training we received was very good. People appreciate my work when I give them talks… (Peer supporter) The referral card…have a look (showing the card)…it has my name, and the date of my next clinic … at the clinic I just showed it (to the staff) and I was attended (FGD participant who attended clinic) I still have my referral card … I never leave it behind…so I know that the day I go to the eye clinic I will show it and get checked (FGD participant who did not attend clinic)
Complexity	Training the peer supporters was not difficult, the training slides are easy to use (trainer) We are not doing things that are very different from what is usually done …[in DSGs]…we were already familiar with group talks, what we have not been doing was the telephone reminders...and giving referrals, but that is not burdensome (peer supporter) When I learnt the reason for the test, and was given the referral card…all I needed was to present myself at the clinic. You could go even the following day, anytime…and you only needed to go once. We went together several of us. Can anyone say that it is difficult? (FGD participant who had screening)
Adaptability and flexibility	I wanted the PLWD to go to the eye clinic as soon as possible…so if I could not reach them on phone because they had put off the phone, I took it upon myself to go to their homes (peer supporter) We carry out the DURE activities because they fit well with our other activities…I go on with my usual work on the farm except for the DSG meetings (peer supporters) During recruitment, we realized that we needed additional personnel in the recruitment team, so we expanded the team (member of research team)
Cost	The intervention uses existing resources in the community and in the hospital…this is the biggest advantage because it can scaled up without cost limitations*…*(MoH county representative) People’s pockets are different…when I went there I had only 100 shillings … I paid 50 for registration. They asked me to pay another 50 for eye examination. I paid it because I had to get the screening. But someone else will just say they will come next week. (FGD participant who attended screening)
Relative disadvantage	The only problem is that at both the diabetes clinic and the eye clinic they make you wait… queuing two times…then in the eye clinic they put some medicine in your eyes and ask you to wait again…you can end of wasting a lot of time waiting. Why should I que twice? (FGD participant who attended screening) But why don’t you focus on preventing the complications, rather than just screening? For me I have begun with doing exercises, but later I will go for the screening. [names peer supporter] will take me there… (FGD participant who did not attend screening)
Outer setting	
Diabetes as a health priority	We are keen on sustainable interventions for NCDs and we have to go to where the patients are found…We should not wait for patients to come but go to them. This is sustainable because the trained peer educators will remain in the DSG to educate more people. We will work with them more. (MoH representative at national level)
Clinical guidelines for DR are used as a national governance tool that is also useful for resource mobilization	The clinical guidelines have been very helpful. Earlier on I did not routinely screen those who have good vision. Now I dilate and screen all those that come here. We also order more dilating drops (eye health worker)
Peer supporters mitigated potential implementation challenges such as political events	The presidential elections were nullified, everyone left the (DSG) meeting to go and watch the news… and had I not been passionate to mobilise members there would have been very poor attendance at the next meeting… (peer supporter)
Intervention fits within the norms of the health care system	The county health services, including eye care and diabetes care services supported this innovative involvement of peer supporters because we all want to improve quality of life for PLWD (MoH representative at county level)
External outreach camps	There were two external mobile outreach camps organized at a church by a private care provider…some of the people preferred to go for the screening here because it was nearer (DSG county lead) With the mobile outreach clinic, you know it is only for one day, so you don’t want to miss the opportunity. For the hospital, some of the people, even if they live near, do not attend… They keep on postponing because the eye clinic will always be here… (Peer supporter)
Inner setting	
Tension for change	I know someone who doesn’t go out of his home now, because he can’t see…that is why we have been told not to wait till we have eye problems (FGD participant who took screening) I have never had my eyes checked…. Can you check me today? Or give us the referral cards so we can go [to the eye clinic] tomorrow (FGD participant from control arm) We have to find a way of easily identifying those any diabetes patient who has not been screened. May be label their files so that they can easily identified (eye health worker) We have seen people going blind…nobody will come from outside to stop it… we have to do something ourselves (KDDA county representative)
Compatibility	We give the group talks as part of the monthly DSG meetings (peer supporter) We want to have all PLWD screened for DR, thus this intervention is contributing to that mandate (eye care worker)
DSG Organisational Culture	For us we are always open to new things that can help us who live with diabetes, so we are happy to work with you on this…it empowers us to not just to go for screening, but to engage in advocacy for diabetes eye health (KDDA local representative) In DSGs, we know about volunteering … and for the good of our people, we all have to work together to ensure everyone goes for the eye check… I do not mind giving my time to do this, though of course it requires extra time … I am happy people got tested (peer supporter) At the DSG I tell them my experience with screening…we don’t hide things from one another…(PLWD who has taken screening) In some of the DSGs, participants came together…we would have a large group turn up at one go…they would tell me they all agreed to come together (eye health worker) Here we like to share about ourselves openly, we don’t hide things, we are not afraid to open up or keep reminding one another about attending screening (FGD participant who attended screening)
Incentives and rewards	We do not get paid for this work, it is about volunteer work, people who do not want to volunteer their time cannot do this work (peer supporter) But since we are doing good work, and we spend a lot of time on it, if we were paid we could do even more (peer supporter)
Readiness for implementation	We are planning a peer supporter training in [names county]…we want you to come and train them so that they start doing the same in [names county]… (KDDA national representative) Now that you have done this with some groups, you also have to come to our groups and give us the intervention, … you should not leave us out (PLWD from control group)
Adjustments in the eye health system	Sometimes, people did not screen for diabetic retinopathy if the patient’s vision was good. But now we have been reminded to screen all PLWD annually and we have started doing that (eye health worker) The eye clinic has recently been renovated, we saw the governor launch it and we heard that it has all the equipment, so we are now happy to go there. (FGD participant who attended screening)
Community volunteers (CVs) reinforced key messages	Community volunteers really support us…because they reinforce what we say. In our DSG, we have a member who is a community volunteer… I usually call her to speak after I given the group talk...it is better when the message comes from two people. (peer supporter)
Geographical barriers hinder uptake of screening	Getting to the eye clinic is a problem because the easiest way is to take a *boda boda* (motorbike taxi) to the main road and then wait for a *matatu* (public van). I avoid *boda boda* because I have a back problem, so I just wait for the outreach camp*.* (FGD participant who has not attended screening) From this experience, the cost of mobility must be borne by the provider, not the PLWD. We must find a way of going to the DSG for screening, rather than asking them to come*.* (member of the steering team) For me, I haven’t gone to the eye clinic. I am looking for the fare. Why can’t you come to do the test here? (FGD participant who has not attended screening)
Peer supporter characteristics	
Knowledge and beliefs about the intervention	The peer supporter at [DSG] informed me that they have started a WhatsApp group for peer supporters…to discuss how they can do more to prevent diabetes complications in general…they feel the work they are doing with DURE can be expanded (PI field notes) I observed that the peer supporters enjoyed giving the group talks, and the key messages were easy to explain and it was a social activity, unlike the paper work which was more of an individual task. They still did the documentation since they were trained to do it. (research assistant)
Individual identification with the role of PS	In our support group, most people have gone to the eye clinic, because [names peer supporter] is very active, and he makes us laugh when he is giving the talk…you cannot get tired…and every time he meets you he will remind you, even at church… (FGD participant who attended clinic) I always see [name] here in the diabetes clinic, bringing his DSG members. Then he also takes them to the eye clinic. Sometimes they tell him they do not have the money for the hospital fee but he insists and they pay (diabetes care worker)
Individual stage of change	All the peer supporters had already taken screening so they must have been good role models (KDDA national representative) None of the peer supporters had any previous training on delivering a diabetes eye health intervention, you could tell that they liked it…the novelty of the information seems to have been a motivator… (Trainer)
Personal attributes	“I did not do as much work as [name], though he and I are the supporters in our group. But he did very well…you know he is younger, ‘*sharp sharp*’ (slang for exuberance) and men can do this work more easily…” (Female peer supporter) [Name] is ever punctual so we know (DSG) meetings will run on time. She is a teacher so she explains very well. That is why many people don’t miss the meetings, and most of us got tested the very first month (FGD participant who attended screening) What I have seen, is that he [peer supporter] is self-sacrificing…from the heart … he closes his business of selling clothes to bring PLWD to both the diabetes clinic and the eye clinic (diabetes clinician) We did not know whether keeping the peer supporters engaged over six months would be challenging…I would say selection of peer supporters is important as they have to be highly motivated and committed (member of steering team)
Self-efficacy of peer supporters	He took me to the eye clinic, together with others… he did not feel bothered about waiting in the queue with us… (FGD participant who attended screening) When I saw that the first five members had gone for screening right after I gave the first talk, I knew I was doing it right, I felt motivated me to continue with the work (peer supporter) When I observed [names peer supporters giving the talk], they performed so well, they answered all the questions. I think it is because they were trained well… (research assistant)
Process of implementation	
Planning	We were very happy to be involved from the beginning…we have always insisted on being involved as equal partners in things that concern us, so we participated (KDDA national representative) I remember the meeting we had at the beginning…when our chairman of KDDA came and introduced the project…we agreed to support… (peer supporter)
Engaging	The research team was really committed…they were always available and we worked so well together, it made us not to leave the work half- way (peer supporter)…we even took lots of photos The community volunteers, they really embrace us…we support one another in the work (peer supporter) I looked forward to the call from the PI every week – it gave me motivation (peer supporter) Regular briefing helped us to keep involved (steering committee member)
Executing	We were of course concerned about the feasibility of the intervention since we have not used the DSG platform before. But we had success with the pilot trial, so this proved not to be a major issue (member of project steering group) The DSGs in the control arm are left out, but we have understood that they can get the intervention thereafter (peer supporter)
Evaluating	I am looking forward to the findings of the study (research nurse) You need to give us a copy of the results…(MoH county representative) We will organize a forum to share the results with the stakeholders (MoH national representative)

*DSG* Diabetes Support Group, *DURE* Uptake of Retinal Examination in Diabetes study, *FGD* Focus Group Discussion, *KDDA* Kenya Defeat Diabetes Association, *MoH* Ministry of Health, *PI* Principal Investigator

#### DURE intervention characteristics

Stakeholders considered the peer-supporter-led intervention to have relative advantage, as it was not feasible to have health workers go to the community to give the same intervention. All PLWD also perceived the DURE intervention as a relative advantage compared with the usual practice where they were not offered screening. In particular, participants found the referral card highly valuable as it represented personalized care and was perceived to make it easier to navigate interaction with eye care providers. Peer supporters valued the training and task shifting which gave them confidence and recognition that they did not have before.

The intervention components were easy to implement along with the usual duties of peer supporters, and were adaptable to suit local needs (such as additional face-to-face reminders for participants who could not be reached by phone). Although research assistants observed that documentation tasks were time-consuming for peer supporters, these peer supporters did not perceive it as difficult. Given that the pilot trial had provided an opportunity to test-drive the intervention, all stakeholders recognized the intervention as “fit for trial”.

Stakeholders noted that the intervention utilized existing resources, rather than requiring additional resources, which pointed to the advantage of cost-efficiency and possibility for scaling up. At the individual level, some PLWD indicated an inability or unwillingness to pay the hospital consultation fee at the eye clinic might be a financial barrier to screening. Another relative disadvantage was the need for queuing at the eye clinic, especially because those who attended the diabetes clinic on the same day had to queue twice. One participant expressed skepticism about prioritizing screening rather than lifestyle interventions (such as physical exercise).

#### Outer setting

Stakeholders highlighted the growing health priority given to diabetes and other non-communicable diseases within national health policy framework as an important outer setting construct that increased stakeholder interest in the implementation of the DURE intervention. The intervention was also perceived to be responding to patients’ need for a patient-centered approach to care, in addition to being aligned with the norms of the health system such as increasing efficiency and access to services.

The recent implementation of the clinical guidelines for DR had sensitized the diabetes and eye health workers that all PLWD need annual screening for DR. Eye care providers also found the guidelines useful for mobilizing required resources such as mydriatic eye drops. The guidelines were considered an aid to implementing the intervention.

Disruptions in the sociopolitical environment presented a potential outer setting constraint. During the study period, there was a disputed election that was subsequently nullified and had to be repeated. It can be challenging to maintain participant attendance to DSG meetings or to screening during periods of political turmoil. The peer supporters mitigated this potential disruption through persuasive communication with participants. The research team similarly maintained communication with all the stakeholders to ensure continued engagement, fidelity, and availability of screening at the eye clinic.

There were two external outreach eye camps in the county during the study period. Some participants attended screening at these camps instead of the eye clinic. This is because they offered the advantage of proximity, convenience, and the perception of being a scarce but valuable opportunity for screening, which constituted an external incentive. As they used the same screening guidelines, any participants screened at the outreach site was taken to have the outcome of interest.

#### Inner setting

The implementation climate is a key sub-construct within the inner setting construct that was found to be associated with the DURE implementation. Among the peer supporters, there was tension for change that resulted from the training, since they perceived that PLWD are vulnerable to vision loss from DR. Similarly, eye care workers expressed the need to detect and treat DR in a timely manner, as most patients presented with advanced DR. The PLWD reported having taken up screening early in the intervention because the group talks raised awareness about their vulnerability to DR, thus raising the relative priority of taking up screening.

Peer supporters found the intervention to have high compatibility as it was seen to respond to the tension for change, and it was designed to fit within the usual support group activities. To this extent, the compatibility was a facilitator for implementation. Participants tended to turn up at the eye clinic in groups especially early in the trial, and since screening procedures take time, there was a risk of overloading the health system. However, we recognized that this potential implementation barrier was foreseeably transient, since the participants would get individualized appointments for subsequent screening. Based on this premise, the eye care workers supported the implementation, and thus the potential challenge transitioned to become an enabler. This theme also emerged in relation to acceptability (above).

The organizational culture of DSGs is another sub-construct that influenced the implementation. One of the participants referenced that DSGs are usually receptive for “new things” especially those that empower the PLWD. Participants pointed to the culture of self-disclosure, which was associated with the willingness of participants to update the peer supporter and DSG members on whether they had taken up screening. Further, the culture of collective action led participants in a DSG to team up and go together for screening. Due to the culture of volunteerism, peer supporters were willing to commit time to deliver the intervention and sometimes to accompany the participants to the eye clinic. However, a potential threat to volunteerism was also voiced by peer supporters who expressed that they could be doing other things (opportunity cost) and that since their work was effective they should receive some incentives from the government.

Readiness for implementation was epitomized by the interest of DSG leadership at national level to begin scale up of peer-supporter training and to incorporate the DURE training curriculum in the peer-supporter training manual. Participants referenced national-level stakeholder interest to scale up the intervention to other counties. On the other hand, eye care workers articulated the need to equip the eye clinic with more staff and technology for screening.

Participants in the control arm requested for the intervention to be implemented in their own DSGs as they felt left out. This was already planned to meet the ethical obligation to ensure that control groups also benefit from the intervention [18]. This implementation in control arm DSGs was implemented subsequent to the trial.

A potential barrier in the structural characteristics construct of the inner setting is the geographical terrain and distance to the eye clinic for geographically remote participants—distance, unsuitable transport options, cost, and time for travel were noted to be challenges for participants of some DSGs.

#### Characteristics of peer supporters

On the sub-construct of knowledge and beliefs about the intervention, all peer supporters attended the training and rated it as a very useful learning experience because they had no previous exposure to eye health. The training was articulated as a facilitator of implementation since it created self-efficacy to deliver the intervention and to answer any questions from participants. Both the training and the task-shifting inherent to the intervention were valued as sources of personal fulfilment and increased role-recognition by peers and health workers. However, supporters identified that their contribution in the intervention risked being overlooked or under-recognized as they had not received formal recognition such as certificates. Providing certificates was a challenge at this stage because we needed to have the training curriculum formally adopted before certification. Providing certificates may facilitate implementation in scale up of the intervention, by stemming potential burnout or turnover of peer supporters.

Regarding individual stage of change, peer supporters expressed a collective (rather than individual) sense of accountability to implement the intervention: “we are the people to make a contribution to reducing the number of people going blind”. This may relate to the culture of collective action, a theme already highlighted under inner setting constructs. Being volunteers, their motivation came from a sense of altruism, but they were also motivated by the training and subsequent initial success of seeing participants attend the screening. The 13/14 peer supporters remained engaged with the trial until the end, and they reported that the weekly telephone contact with the principal investigator kept them engaged. Community volunteers were a further source of motivation, even though they do not have diabetes. This is because they are also volunteers in the community, are well versed with the health system and they reinforced the key messages.

Cultural adaptation was further highlighted at the peer supporter level, especially relating to gender and age norms in the performance of peer support roles. For example, a female peer supporter noted that she took on a smaller proportion of the tasks and left the rest to her younger male counterpart. However, age and gender were not sufficient to account for the effectiveness of peer supporters. Participants identified some personal attributes of peer supporters that influenced them to take up screening. These included interpersonal skills demonstrated while delivering the group talk (e.g., using humour, keeping time), persistence with follow-up, individual reminders and accompanying participants to the eye clinic.

#### Process

Collaborating with stakeholders emerged as an important attribute. DSG leadership at national and county level were satisfied that they had been involved in the planning of the implementation, through attending pre-implementation meetings. They also noted that the community entry process (through the national, county, and support group leadership) positively influenced acceptability of the intervention.

Continuous engagement was considered critical, in the form of frequent telephone contact or face-to-face contact with all the stakeholders. Peer supporters found the training and weekly telephone calls to give them a sense of identity with the trial. Research assistants valued role modelling for their tasks, while all stakeholders valued regular feedback. In the executing sub-construct, the trial was executed as per the trial protocol. It was not feasible to mask the intervention arm to the intervention, since the intervention activities within a DSG were overt. However, we did not find any evidence of contamination between clusters, perhaps because participants were not privy to the intervention allocation of other DSGs. Regarding reflecting and evaluating, we regularly reviewed the progress and quality of implementation with peer supporter and eye care workers. Peer supporters constantly reflected on their own performance and gave updates particularly on the reach and fidelity. All key informants and participants looked forward to receiving the results of the trial.

### Over-arching analysis of the factors influencing implementation

**Fig. 3:**
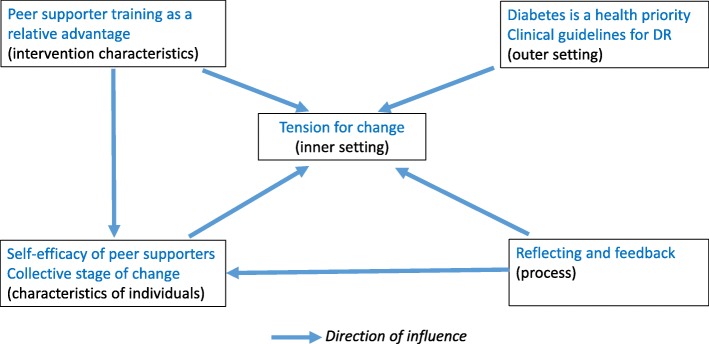
Inter-relatedness of the CFIR constructs

We found that the intervention was in alignment with health system priorities and stakeholder interest. All five constructs of the CFIR impacted the extent to which the intervention was implemented, and the sub-constructs within different constructs were inter-related. For example, among the participants, tension for change developed when the intervention raised awareness of the vulnerability to vision loss from DR. Training of peer supporters enabled them to deliver the intervention, as well as to develop self-efficacy to increase uptake of screening among participants. Getting feedback on the number of people who had attended screening further increased self-efficacy among peer supporters. Prioritization of diabetes and implementation of clinical guidelines for DR also created tension for change among health workers who conducted the screening. Figure 3 illustrates this example of inter-relatedness. Most of the factors identified in each of the constructs were facilitators of implementation, and were modifiable, which means interventions targeting these factors can improve implementation during scaling up.

## Discussion

The DURE study process evaluation provides detailed information on the implementation outcomes and the context of implementation to better understand how the intervention and context interacted. This is the first trial to increase access to DR screening through peer support interventions that has also documented the implementation process. The strengths of this study include the use of a validated theory (CFIR). This theory focuses on broad constructs that are representative of the potential influences on the implementation process, which enhances the generalizability and replicability of our methods [12]. We collected data from different stakeholders, which provided an understanding of how different stakeholders experienced the intervention and its implementation strategies. We engaged PLWD who had gone for screening as well as those who had not to identify different perspectives on facilitators and enablers for the intervention. We collected extensive data using mixed-methods, and we triangulated findings from different sources for validity and to reduce the potential risk of bias. The qualitative data were in broad agreement and provided rich context for the quantitative findings, which enhances confidence in the findings.

The trial had success with implementation outcomes, i.e., acceptability, recruitment and retention, reach, fidelity, and dose. This success resulted from the perception of stakeholders and that the intervention was beneficial. The stakeholder interest, the tension for change and the increasing priority given to diabetes in the national health policy suggest that this was an opportune time for the intervention.

Stakeholders identified that the national clinical guidelines for DR positively influence the practice and readiness of eye clinics to provide screening. By sensitizing health workers to the need to screen all PLWD, the guidelines created tension for change. This in turn ensured that all who turned up for screening received the service, as it addressed potential supply-side barriers to screening. We therefore conclude that health system strengthening such as the development and implementation of clinical guidelines was a facilitator for the implementation of the intervention as well as adoption of screening behaviour by the participants. Several studies have found that health system strengthening gives traction to heath care interventions [18–21].

We observed an early response to the intervention activities. The highest attendance at group talks was at the first group talk. In addition, all participants were referred to the eye clinic for DR screening at the start of the trial. Participants who took up screening reported doing so as soon as they received the group talk and referral, and other DSG members accompanied them. This may be related to the influence of the culture of collective action as well as the effect of the group talk and referral card. We therefore hypothesise that the first group talk and the referral are the most essential components of the intervention. We also hypothesise that these components are well aligned to the causes of non-attendance to screening in this population, which we had found to be inadequate knowledge of diabetes eye complications and lack of referral of referral for screening [2].

Among those in the intervention arm who did not take up screening in the intervention arm, lack of knowledge was not identified as a barrier, suggesting that the intervention had good reach and had increased awareness even among those who did not take up screening. At the same time, participants who did not attend screening still indicated intention to take up screening. These points to the need to identify the post-awareness/intention barriers among PLWD. Grimshaw et al. (2014) had a similar finding among physicians who received an educational intervention to increase referrals of PLWD for DR screening [12]. There were some potential relative disadvantages of the intervention, such as having to queue and pay consultation fee twice if a PLWD was attending both diabetes and eye clinics. This was foreseeable, given the structure of the hospital. These individual, social, and organizational level barriers are consistent with those in the literature [12, 21–23]. Some hospitals are testing the effectiveness of conducting DR screening at the diabetes clinic to overcome this challenge [24]. There is also need to support geographically remote individuals with logistical and financial challenges, such as through multiple access points for DR screening, closer to home [25].

The role of peer supporters in delivering the intervention was a prominent facilitator. Peer supporters delivered the health education talks and telephone reminders, but in addition, they visited participants who were not reachable on phone, accompanied participants to the eye clinics, and waited with them. These are roles of peer supporters that we had envisaged would contribute to increasing the self-efficacy of participants who took up screening [3]. This is evidence that the intervention worked through the anticipated mechanism of the intervention, which strengthens the plausibility of the results [26]. The peer supporters also identified the training and task shifting as very valuable, and these were important enablers for the role of peer supporters. We also found that community volunteers to be an important enabler to peer supporter roles, which we had not anticipated. This is evidence that complex interventions interact with the context in multiple ways to influence the outcomes.

The intervention was compatible with processes and activities of both the DSGs and the eye clinics. We noted adaptation to the delivery of the intervention (but not to the content of the intervention), driven by contextual experience. Like other investigators of pragmatic public health interventions, we recognized the need for fidelity-adaptation balance, since they coexist and are both valuable [10, 27]. To mitigate the risk of intervention drift that may occur with adaptation, we monitored the level of fidelity, which was maintained. Rather than threatening fidelity, we found that this adaptation actually maintained fidelity, because it ensured that those who could not be reached by phone still got the scheduled reminders to attend screening.

Our findings highlight that all the constructs of the CFIR were relevant to the trial, and there were multiple determinants to implementation. We cannot attribute the success of implementation exclusively to the characteristics of the intervention, the peer supporters, the DSGs, eye clinics, the outer setting, or implementation processes. More likely, it is the combination and interconnectivity of these constructs working together. This highlights the importance of alignment of interventions to context.

The process evaluation has potential limitations. We did not conduct evaluation at multiple points in the trial; hence, we could not capture changes over time. As the study was conducted in multiple DSGs with different peer supporters, we cannot account for differences in peer support styles. The social and health system context of the trial may also affect the generalizability of its results. The potential for researcher bias by the project investigator undertaking the qualitative interviews is acknowledged; however, it was agreed among the project team that she was the most culturally appropriate person to probe about how the context affected implementation and the mechanisms of impact. We cannot rule out response bias by social desirability, but the risk was low, given that this was not a sensitive topic, and we relied on diverse sources of data.

## Conclusion

The intervention was largely implemented as designed, and achieved high implementation outcomes for acceptability, recruitment, retention, reach, fidelity, and dose. There is high stakeholder interest to support scale up. The intervention worked through the expected mechanisms, but was also aided by unanticipated mechanisms. Health system strengthening was a necessary pre-requisite for implementation. We recommend that future process evaluations are carried out at multiple points and include a cost analysis.

## Supplementary information

**Additional file 1.** Methodology for development of training for peer supporters

## Data Availability

The data that support the findings of this study are available from Ministry of Health, Kenya but restrictions apply to the availability of these data, which were used under license for the current study, and so are not publicly available. Data are however available from the authors upon reasonable request and with permission of the Ministry of Health.

## Abbreviations

CFIR: Consolidated Framework for Implementation Research
cRCT: Cluster Randomized Controlled Trial
DR: Diabetic Retinopathy
DSG: Diabetes Support Group
DURE: Uptake of Retinal Examination in Diabetes study
FGD: Focus Group Discussion
KDDA: Kenya Defeat Diabetes Association
PLWD: People living with diabetes
