# Supplementary material for: Peer-support to increase uptake of screening for diabetic retinopathy: process evaluation of the DURE cluster randomized trial
Source: Trop Med Health. 2020 Jan 6;48:1. doi: 10.1186/s41182-019-0188-z (PMC6945600; doi:10.1186/s41182-019-0188-z)
Supplement: Supplementary file 1 — Additional file 1. Methodology for development of training for peer supporters [file 41182_2019_188_MOESM1_ESM.docx]

## Methodology for developing the competencies and training content

Context: The content of the training is incremental to the existing training manual for peer supporters.

### Process

The following steps were completed:

1. Identification of a local gap. The health system assessment for diabetes and diabetic retinopathy identified that uptake of eye examination in three counties is low. A peer-led educational and referral intervention aims to address the demand side barriers to uptake of eye examination.
2. Literature review of peer support: showed that peer support has been effective in contributing to education of PLWD and linkage with health care resources.
3. A review of the existing peer support training manual has been conducted – we noted that it does not have content on diabetes eye health.
4. Literature review on development of training programs identified the factors associated with greater impact of training initiatives to be: alignment to local needs and priorities, country ownership of the training program, competency-based training, and a sustainability strategy.
5. Competencies for peer supporters have been developed, based on the literature and the identified local need
6. Assessment of the social accountability of this training. Social accountability in medical education is defined as an education that responds to the requirements and expectations of the society. This training has been assessed for relevance, cost-effectiveness, quality and equity.
7. Relevance: The training is relevant because it addresses a need that has been identified in this community.
8. Cost-effectiveness: The training is cost-effective because it is low cost in terms of training resource. It requires only 2 days and hence it can easily be added to the existing training without causing significant disruption to the training arrangements during the scale up phase. In addition, if necessary a shorter version of the training can also be created.
9. Quality Assurance: The identification of concise competencies provides a consistent standard of knowledge and skill. The training will also consist of structured practical sessions and role modelling to aid application of knowledge.
10. Equity: The training contributes to enhancing equity because it empowers disadvantaged individuals and groups to obtain the eye examination that they require.
11. Stakeholder consultation: The draft competencies were reviewed by ophthalmologists, endocrinologists, physicians, diabetes educators, public eye health specialists and the umbrella body for peer supporters (Kenya Defeat Diabetes Association).
12. The curriculum was presented at a local workshop and a local conference for discussion. This was done to foster a sense of ownership among the stakeholders.
